# Case for omitting tied observations in the two-sample t-test and the Wilcoxon-Mann-Whitney Test

**DOI:** 10.1371/journal.pone.0200837

**Published:** 2018-07-24

**Authors:** Monnie McGee

**Affiliations:** Department of Statistical Science, Southern Methodist University, Dallas, Texas, United States of America; University of Kentucky, UNITED STATES

## Abstract

When the distributional assumptions for a t-test are not met, the default position of many analysts is to resort to a rank-based test, such as the Wilcoxon-Mann-Whitney Test to compare the difference in means between two samples. The Wilcoxon-Mann-Whitney Test presents no danger of tied observations when the observations in the data are continuous. However, in practice, observations are discretized due various logical reasons, or the data are ordinal in nature. When ranks are tied, most textbooks recommend using mid-ranks to replace the tied ranks, a practice that affects the distribution of the Wilcoxon-Mann-Whitney Test under the null hypothesis. Other methods for breaking ties have also been proposed. In this study, we examine four tie-breaking methods—average-scores, mid-ranks, jittering, and omission—for their effects on Type I and Type II error of the Wilcoxon-Mann-Whitney Test and the two-sample t-test for various combinations of sample sizes, underlying population distributions, and percentages of tied observations. We use the results to determine the maximum percentage of ties for which the power and size are seriously affected, and for which method of tie-breaking results in the best Type I and Type II error properties. Not surprisingly, the underlying population distribution of the data has less of an effect on the Wilcoxon-Mann-Whitney Test than on the t-test. Surprisingly, we find that the jittering and omission methods tend to hold Type I error at the nominal level, even for small sample sizes, with no substantial sacrifice in terms of Type II error. Furthermore, the t-test and the Wilcoxon-Mann-Whitney Test are equally effected by ties in terms of Type I and Type II error; therefore, we recommend omitting tied observations when they occur for both the two-sample t-test and the Wilcoxon-Mann-Whitney due to the bias in Type I error that is created when tied observations are left in the data, in the case of the t-test, or adjusted using mid-ranks or average-scores, in the case of the Wilcoxon-Mann-Whitney.

## Introduction

A common experimental design is to compare two independent samples. Independent samples are such that the observations pertaining to each sample have been randomly selected from two different populations. For example, a researcher might compare the amount of tritiated water diffusion across the placental membrane in pregnancies ending at 12-26 weeks gestational age and full-term pregnancies [1]. The measurements for tritiated water on five early-term and 10 full-term pregnancies are given in [2]. For the purposes of the statistical test, the five pregnancies ending at 12-26 weeks gestation are considered a random sample of size five from the population of early-term pregnancies, and the other 10 pregnancies are considered a random sample of size 10 from the population of full-term pregnancies. Obviously, no relationship exists between the populations. This clear delineation in population membership is what is meant by “independent” samples.

The two most popular statistical tests for such a scenario are the two-sample t-test [3] (also called the independent samples t-test) and the Wilcoxon-Mann-Whitney Test (WMW), also called the Mann-Whitney U Test or the Wilcoxon Rank Sum Test [4] [5]. The two-sample t-test (TST) is a parametric test, which means that the numeric observations collected for the test are assumed to have been drawn from a specific theoretical distribution. In the case of the TST, the underlying population distribution is the Normal distribution.

More formally, let *X* = *X*_1_, *X*_2_, …, *X*_*n*_ be a random sample from a Normally distributed population with mean *μ*_*X*_ and variance $\sigma_{X}^{2}$, and *Y* = *Y*_1_, *Y*_2_, …, *Y*_*m*_ be a random sample from a Normally distributed population with mean *μ*_*Y*_ and variance $\sigma_{Y}^{2}$. For simplicity, the population variances of the Normal distributions are assumed to be equivalent, that is $\sigma_{X}^{2}=\sigma_{Y}^{2}$; however, in practice, different versions of the TST relax this assumption (i.e. Welch’s t-test [6]). The TST has the hypotheses that

$$\begin{array}{c} H_{0}:\;\mu_{X}=\mu_{Y}\\ H_{A}:\;\mu_{X}\neq \mu_{Y} \end{array}$$ (1)

with test statistic (when population standard deviations are unequal)

$$\begin{array}{c} t=\frac{\left(\bar{x}-\bar{y})-(\mu_{X}-\mu_{Y}\right)}{\sqrt{\frac{s_{X}^{2}}{n}+\frac{s_{y}^{2}}{m}}} \end{array}$$ (2)

here, $\bar{x}$ and $\bar{y}$ are the sample means for *X* and *Y*, respectively, and *s*_*X*_ and *s*_*Y*_ are the respective sample standard deviations. Under the null hypothesis, most of the time *μ*_*X*_ − *μ*_*Y*_ = 0.

The Wilcoxon-Mann-Whitney Test (WMW) is a nonparametric alternative to the TST. It is nonparametric in the sense that the functional form of the population distributions from which the data are drawn is not specified, but the lack of specification of a particular population distribution from which the data are sampled does not imply that the test has no assumptions. First, in the original formulation of the WMW [4], it is assumed that the data are drawn from a continuous distribution. This assumption is implicit in the parametric assumption of a Normal distribution for the TST. Second, the two population distributions are also assumed to have the same general shape, although the shape itself is not specified [7].

In Wilcoxon’s formulation of the test [4], the two samples, *X* = *X*_1_, *X*_2_, …, *X*_*n*_ and *Y* = *Y*_1_, *Y*_2_, …, *Y*_*m*_, are combined. Then, the observations are ranked regardless of sample membership. Let the combined sample be denoted as *W* = {*X*_1_, *X*_2_, …, *X*_*n*_, *Y*_1_, *Y*_2_, …, *Y*_*m*_} = {*W*_1_, *W*_2_, …, *W*_*n*+*m*_}. Ranks are given as *r*(*W*_1_), *r*(*W*_2_), …, *r*(*W*_*n*+*m*_), where *r*(*W*_*i*_) is the number of observations *W*_*j*_, *j* = 1, 2, …, *n* + *m*, such that *W*_*j*_ ≤ *W*_*i*_. Once the observations are ranked, the two samples are separated with the corresponding overall ranks attached to each observation. The WMW statistic is given as the sum of the ranks from one of the samples, denoted by $S_{n}=\sum_{i=1}^{n}r\left(W_{i}\right)$, where the *r*(*W*_*i*_) come from the same sample [7].

Tail probabilities for the exact distribution of the WMW, usually for “small” sample sizes up to no more than 15, are given in various tables (*e.g.* Table H of [8]). Also, a Normal approximation exists, given by the following formulation:

$$\begin{array}{c} Z=\frac{S_{n}-n\left(n+m+1\right)/2}{\sqrt{mn(m+n+1)/12}}. \end{array}$$ (3)

The Normal approximation is recommended for “large” sample sizes; however, “large” often is arbitrarily defined. The validity of this approximation is investigated in [9] for combinations of sample sizes where *n* is between 1 and 5 and *m* is between 1 and 8. It was reported that the Normal approximation for the WMW is accurate for sample sizes as small as *n* = *m* = 5 [9]. The authors of [9] did not consider the influence of tied observations.

Most introductory statistics textbooks recommend that the TST is applicable in situations when the underlying population distribution for the data is Normally distributed (or at least symmetric), the sample size is large, and outliers do not exist in the observed sample data [10]. In contrast, for the WMW, current recommendations suggest its use when sample sizes are small because it is difficult to for an analyst to determine whether the form of the underlying population distribution using graphical and numerical descriptive statistics when the sample size is small. The WMW is also recommended when the underlying population distribution is not symmetric, or the observations are discrete [11]. However, one assumption of the WMW test is that the data come from a continuous distribution—a fact that is often ignored when considering the appropriate two sample test to apply to a given set of data. This assumption is important because if data are sampled from a continuous distribution, the probability that any two observations will be equal (leading to tied ranks in the rank-transformed data) is 0. If the data are discrete, then the possibility of tied ranks exists. Therefore, users need to be familiar with standard procedure to deal with tied ranks and with the consequences to Type I and Type II errors of each procedure. A Type I error, also called the *size* of a test, is rejecting the null hypothesis in Eq 1 when it is true. A Type II error is failing to reject the null hypothesis when it is false. In either case, users make an incorrect decision about the hypotheses in Eq 1 from the data. Analysts often refer to the *power* of a test, which is the probability of rejecting the null when it is false, which leads to a correct decision. The power of a test is given by 1—Pr(Type II error).

### Adjustments for tied observations

In practice, observations from continuous data are often discretized or rounded due to the detection limits of measurement instruments, the nature of the phenomenon observed, or simply for convenience. In such situations, some observations may be equal, which means that their ranks will be equal in the case of the WMW. More formally, if the *i*^*th*^ value in a set of *n* + *m* = *N* observations is repeated with frequency *t*_*i*_, *t*_*i*_ ≥ 2, then this observation is a tied observation. Tied observations create problems because there are ∏*t*_*i*_! possible assignments of ranks to the entire sample with ties [8]. Each assignment leads to its own value of the rank test statistic. Some of these values may be equivalent; however, the presence of ties leads to a situation in which the test statistic cannot be computed with the usual tables or via Eq 3.

Several methods exist to adjust for ties in the ranks for the WMW. The following list comes from [8].

*Randomization:* One of the *t*_*i*_, *t*_*i*_ ≥ 2 assignments is selected at random to use as the assignment of the ranks. Each assignment occurs with equal probability, which means that the distribution of the rank order statistic under the null hypothesis is unchanged. However, this method adds an additional (artificial) element of chance to the rank assignments, which affects the distribution under possible alternative hypotheses.

*Midranks:* Using the mid-rank solution, the tied scores are assigned the average rank of the ranks that they would have received had they been distinct. For example, suppose we have the following data that represent a score on a scale of alertness of 11 students, five who had consumed 8 ounces of coffee prior to the test, and six who drank 8 ounces of water. For the coffee group, we have scores 21 31 29 27 35, and for the water group, we have 21 19 17 21 20 19. Instead of using the scores directly, we want to use the ranks of the scores for a nonparametric test. First, we sort the scores to obtain 17 19 19 20 21 21 21 27 29 31 35. Now, we want to assign the ranks 1, 2, 3, …, 11 to the observations; however, there are two sets of scores that are the same: two 19s and three 21s. Using mid-ranks to break the ties, the two 19s would be assigned the rank of 2.5, and the three 21s would be assigned the rank of 6, resulting in the following vector of ranks: 1, 2.5, 2.5, 4, 6, 6, 6, 8, 9, 10, 11.

The mid-rank method is the most widely used, and often is the only method mentioned for dealing with ties when the tied-rank issue is discussed in introductory (and even some more advanced) textbooks [2]. Contrary to the randomization method, the mid-rank method reduces the variance of the null distribution because some ranks will be the same. The magnitude of this change depends on the number of sets of ties (*i*, where *i* = 1, …, *n* + *m*) and the number of ties within each set (*t*_*i*_). However, in the presence of ties, corrections for the variance exist [8]. In addition, the mid-rank method is proven to be asymptotically more efficient than the average statistic method (discussed next) [12].

*Average Statistic:* To avoid the arbitrary nature of choosing one of *t*_*i*_, *t*_*i*_ ≥ 2 possible assignments of ranks, instead compute the value of the rank statistic under all possible combinations (or a large subset of combinations) and use their average value as the rank statistic. This method also decreases the variance under the null hypothesis and reduces asymptotic efficiency [12].

*Average Probability:* A variation on the average statistic method uses each of the possible assignments to obtain the probability of the resulting rank statistic under the null hypothesis. Then, an average of probabilities will obtain the overall probability. For average probability to be valid, the exact null probabilities must be calculated.

*Least Favorable Statistic:* If one has all possible values of the test statistic (or the probabilities), are available, the value that minimizes the probability of rejection (or, correspondingly, the largest probability) could be chosen. This choice is the most conservative of the procedures mentioned in this manuscript.

*Range of Probability:* Another method is to compute the most favorable in conjunction with the least favorable statistic, and to report both values. This method may not lead to a decision about the null hypothesis, because one statistic could lead to values that support the null hypothesis, and the other could lead to probabilities that support the alternative hypothesis.

*Omit Tied Observations:* This method originally was recommended in the context of the sign test for one sample data [13]; it is shown to be the unique most powerful test in this context [12]. Its use carries over to the WMW; however, the literature does not contain an explicit recommendation to omit tied observations for WMW. Certainly, ignoring the ties reduces the sample size, which results in a loss of power. The loss is not too great if “not that many” ties exist. An advantage of the method is that it reduces bias toward rejection of the null hypothesis.

*Jittering:* In this scenario, random noise is added to each data point prior to the ranks being assigned. For example, suppose a small amount of random normal noise is added to the original scores in the alertness data. Then, a possible realization of the jittered data is 21.00052 31.00002 29.00056 26.99977 35.00048 20.99991 19.00031 16.99932 21.00021 19.99977 18.99988. Now, the values are distinct, and can be ranked from 1 to 11 without being adjusted for tied ranks. Jittering is widely used in graphics in order to increase visibility of points on a graph that overlap. In this context, it generally is accepted [14, 15]. Jittering is hardly discussed in the context of hypothesis testing, and it is not been compared with the other methods for size and power considerations.

Some statisticians have expressed distrust in a method that adds random noise to a process that already involves random selection [16]. Another approach allows random tie breaking using jittering, as long as the user realizes that each different application of jittering potentially yields a different rank statistic, and that different statistic necessitates the application of the jittering multiple times [17], as is done with the average score or average probability methods. The multiple jittering approach is applied in this paper. To our knowledge, no one offers a systematic comparison of the effect of jittering on the size and power of the WMW.

### Previous work

Duenas [18] considers the treatment of ties in the two-sample problem, the multiple-sample problem, and in rank correlation tests. She states a result of Dixon and Massey [13], in which the omission of tied observations is recommended, even though the sample size is correspondingly reduced. However, she considers only mid-ranks and random assignment as methods of tie-breaking in the case of the WMW. Her results concur with Putter’s previous results in that the random assignment method leads to reduced efficiency [12]. The mid-ranks method is recommended for small samples. She also cites a correction of Eq 3, given in Eq 4, for the case when mid-ranks are used to handle tied observations [19]. The mean for the Normal approximation is unchanged in this formulation, and the variance for the Normal approximation has the following form:

$$\begin{array}{c} \sigma^{2}\left(U\right)=(\frac{nm}{N(N-1)})(\frac{N^{3}-N}{12-\sum_{i=1}^{g}\frac{t^{3}+t}{12}}), \end{array}$$ (4)

where *t* is the number of observations of a given rank and *g* is the number of groups of ties. Eq 4 is recommended by Siegel [20] if (1) the proportion of ties is large, (2) some of the *t*s are quite large, and (3), the value of Eq 3 corresponds to a probability value that is close to the given critical value.

In considering tie-breaking methods, Fay [21] examines nine different methods and their effects on the Type I error and power on six different distribution-free tests. Four of these tests; the Kolomorgorov-Smirnov Test of general differences [22], Rosenbaum’s Test of location [23], Tukey’s quick test of location [24], and WMW—are tests of location for independent samples data. He also considers tie-breaking methods (including five methods mentioned earlier in this manuscript) as well as four methods that are specific to only one or two tests (not the WMW). Further, he considers the effect of tie-breaking methods using four of the Micceri data sets [25]: extreme asymmetric, extreme bi-modal, multi-modal lumpy, and smooth symmetric. Fay finds that the preferred tie-breaking method for WMW is either randomization or average-scores, depending on the underlying distribution of the data. Fay also examines tie-breaking methods when they are used with Likert-Scale data, a situation in which the WMW is usually recommended because the data are decidedly not Normally distributed. However, Fay finds that none of the explored tests or tie-breaking methods produces a satisfactory Type I error or power. He does not recommend any of combinations of tests and tie-breaking methods for “discrete population data sets than contain relatively few distinct values” [21].

Although many methods exist to break ties in the WMW, the mid-rank method is the most often used. This method is frequently implemented with the function wilcox_test in the R package coin [26, 27]. This function also has an option to use the average-scores method to handle tied ranks. However, this function does not consider jittering. In the current work, Type I and Type II error for jittering are compared to Type I and Type II error for the mid-rank and average-scores methods. When the two samples have different sizes (*n* ≠ *m*), with various values of the ratio between *n* and *m*, simulations are also conducted. Furthermore, we also consider the effect of ties on the TST, which to the present, is not studied with simulations. For both TST and WMW, four underlying population distributions—the Normal, the Cauchy, the Laplace, and the exponential—are studied, as well as the effect of differing percentages of tied observations.

## Materials and methods

In order to calculate the probability of Type I error, *α*, we created 2000 data sets for each two-sample test (TST or WMW) when the null hypothesis is true. In other words, the data sets were created such that *μ*_*X*_ = *μ*_*Y*_. Without loss of generality, *μ*_*X*_ = *μ*_*Y*_ = 0. Under this assumption we varied the sample size (*n* = *m* = 3 to *n* = *m* = 20), underlying population distribution, and percentage of tied observations. This amounts to 2000 different hypothesis tests for each combination of sample size, distribution, etc. We determine *α* for each combination by calculating the number of of p–values less than 0.05 and dividing by 2000. We replicate each scenario100 times and average the calculated p-values. The percentage of tied observations investigated are no ties, 10% ties, 25% ties, and 50% ties. The tied observations are evenly distributed across samples in our simulations: *i.e.* we do not consider the effect of the distribution of the tied observations within the sample itself.

To determine the probability of Type II error, *β* (Power = 1 − *β*), we created 2000 data sets for the same combinations of sample size, underlying population, and percentage of tied observations as was done for the simulations of Type I error. However, to determine Type II error, the alternative hypothesis must be true. Therefore, added a “location shift” (*δ*) of either 0.5, 1, or 1.5 to determine the effect of the amount of the shift on the various combinations. The location shifts are meant to mimic small, medium, and large changes in location, respectively. We did not consider changes in variability. Power was calculated in an analogous manner to the Type I error: we counted the number of times that the p-value for each of the 2000 tests was less than 0.05 (a rejection, which is the correct response) and divided by 2000.

We used one of four methods—omission of ties, mid-ranks, average scores, or jittering—to correct for the tied observations in the WMW. For the TST, the ties were not adjusted because usual practice is not to adjust for ties when performing a t-test. Our investigated sample sizes ranged from 3 to 20 for each group (*i.e.* 6 to 40 total observations), and the simulations are done for both equal and unequal sample sizes. In addition, we draw data from four different theoretical distributions: Normal, exponential, Cauchy, and Laplace. All simulations were done in the open source language R, version 3.4.3 [26], on an iMac with a 3.3 GHz Intel Core i7 processor running OS version 10.11.6.

The R function jitter is used to add random noise to tied observations. The function takes three arguments: the data vector to be jittered, a factor, and an amount. The function adds random uniform noise on the range (−*a*, *a*) to every object in the vector, where *a* = *f* * (*d*/5) by default. The default for the *f*, called the “factor”, is 1, and *d* is the smallest difference between adjacent unique observations in the vector. When the data are jittered, noise is added to only to one of the samples prior to ranking to insure that no ties are present. We repeat this method 100 times and the average p-value is obtained for these scenarios, in order to mitigate the effect of the addition of random noise to one of the samples.

Data values for the TST are the same in each case as those used for the WMW. Clearly, if a large number of tied observations is present, the standard deviation of the data will be smaller than expected, thus causing the t-statistic to be larger than expected, and resulting in an increased rejection rate. Our results show that this diminishes the power of the t-test. Therefore, when tied observations are present, neither the TST nor the WMW performs as well in terms of power or size as when no ties are present.

## Results and discussion

### Type I error and power for the four adjustment scenarios

**Fig 1:**
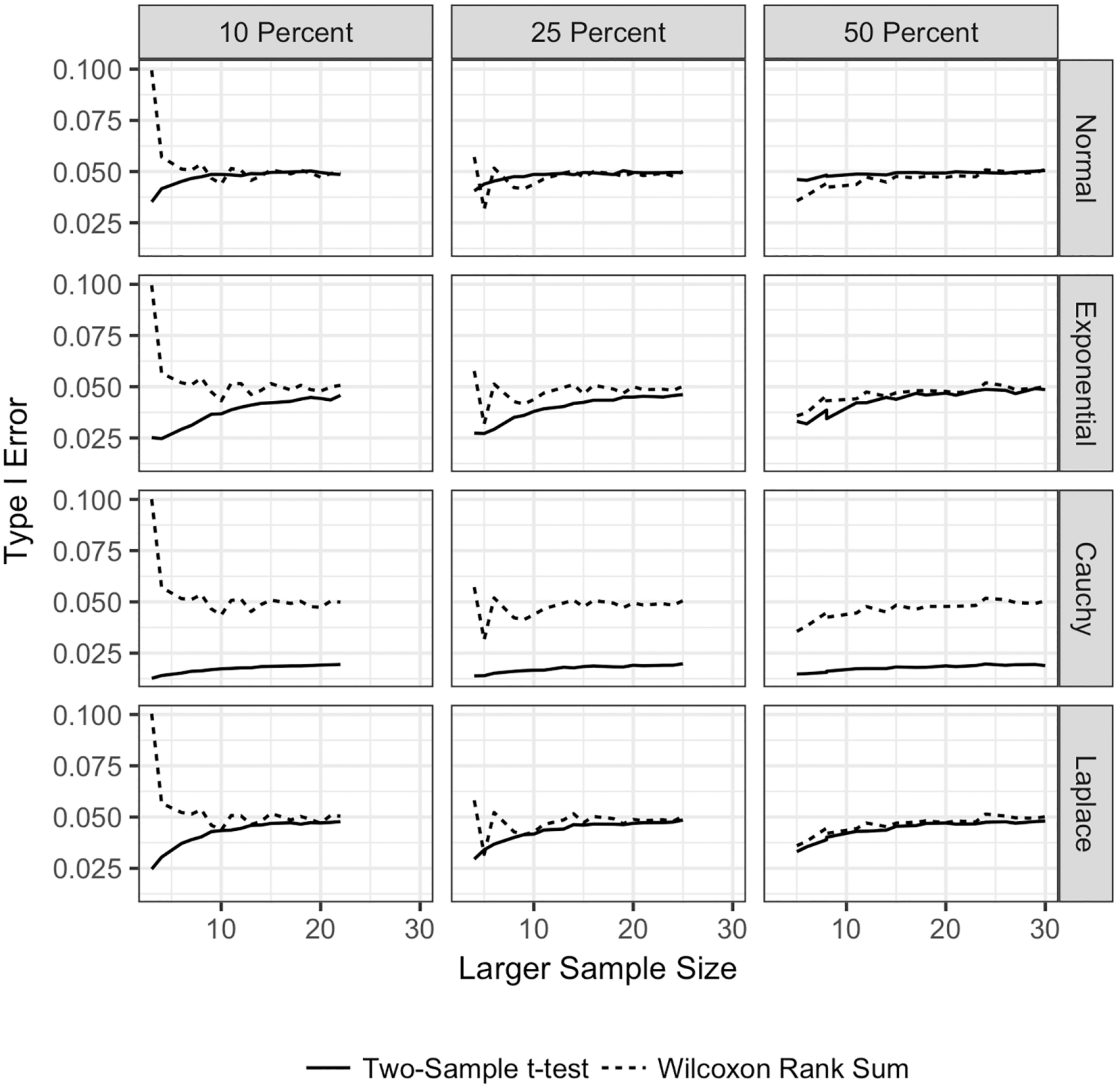
Type I error for the TST and WMW with no ties and unequal sample sizes. Type I error for both the TST and WMW when there are no ties, unequal sample sizes, and continuous distributions. Type I error is plotted on the vertical axis, and the sample size for the larger sample is plotted on the horizontal axis. The Type I error for the TST is plotted with a solid line 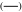, and the Type I error for the WMW is plotted with a dashed line 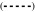. Each row corresponds to a different underlying population distribution: the Normal (top row), exponential (second row), Cauchy (third row) and Laplace (bottom row). Each column corresponds to a different percentage difference between the sample sizes. In the left most column, the larger sample size is 10% larger than the smaller sample size. In the middle column, the larger sample size is 25% larger than the smaller sample size. In the right column, the larger sample size is 50% larger than the smaller sample size. Each frame shows the empirical Type I error under each combination of distribution and sample size difference.

**Fig 2:**
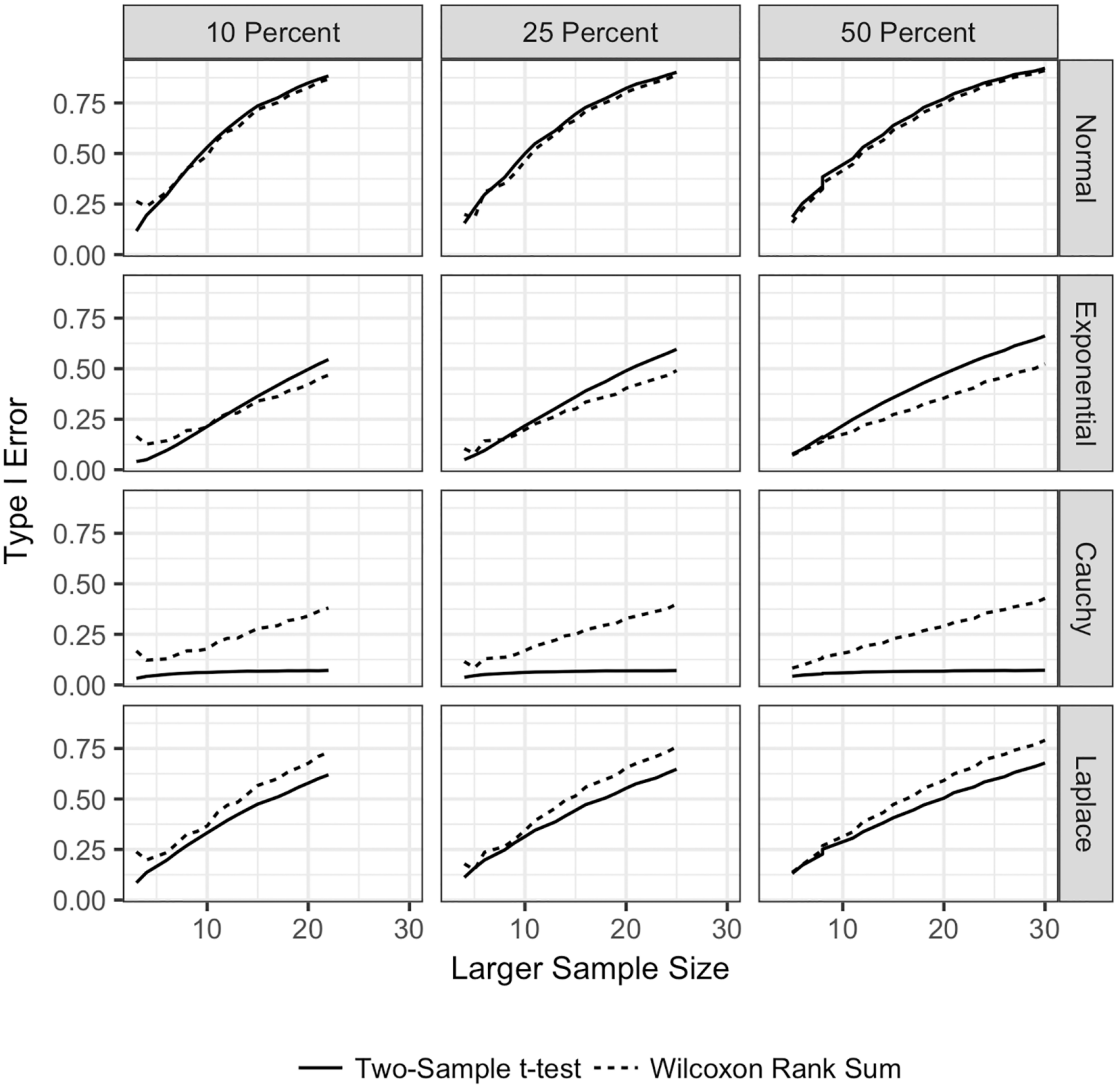
Power for the TST and WMW with no ties and unequal sample sizes. Power for both the TST and WMW when there are no ties, unequal sample sizes, and continuous distributions. Power is plotted on the vertical axis, and the sample size for the larger sample is plotted on the horizontal axis. The power for the TST is plotted with a solid line 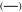, and the power for the WMW is plotted with a dashed line 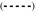. Each row corresponds to a different underlying population distribution: the Normal (top row), exponential (second row), Cauchy (third row) and Laplace (bottom row). Each column corresponds to a different percentage difference between the sample sizes. In the left most column, the larger sample size is 10% larger than the smaller sample size. In the middle column, the larger sample size is 25% larger than the smaller sample size. In the right column, the larger sample size is 50% larger than the smaller sample size. Each frame shows the empirical power under each combination of distribution and sample size difference.

The results of simulations for Type I error and power for unequal sample sizes when ties are not present are plotted Figs 1 and 2. Type I error and power versus the sample size for the TST and WMW under the scenario of equal sample sizes with no ties in the data are given for comparison purposes in S1 and S2 Figs.

Fig 1 shows the results for Type I error in the case where the sample sizes for the two groups are unequal. We see that the TST is more affected than the WMW by the underlying distribution of the data, as has been previously shown for data when the sample sizes for the two groups are equal [11]. In Fig 1, we plot the larger of the two sample sizes on the horizontal axis for each frame. Each column represents a change in the percentage difference between the sample sizes. In the left column, the larger sample size is 10% larger than the smaller sample size; similarly for the other two columns. Reading across the columns, we see that as the percentage difference between the sample sizes increases, the Type I error decreases from 0.1 to the nominal level of 0.05 for the WMW, regardless of the distribution. However, the Type I error for the TST increases from approximately 0.025 to the nominal level except for the Cauchy distribution. The Type I error for the WMW never goes below 0.037, whereas the Type I error for the TST in this situation can be below 0.02 for the Cauchy distribution and close to 0.03 for the Laplace and exponential distributions. The main conclusion from this figure is that, except for very small sample sizes, the WMW maintains the nominal Type I error level regardless of the distribution, whereas the TST can be quite conservative for all sample sizes, particularly in the presence of outliers (as represented by the Cauchy distribution).

Fig 2 shows the power for the TST and WMW for unequal sample sizes when there are no ties in the data and *δ* = 1. For *δ* = 0.5, neither test had reasonable power for any sample size, and for *δ* = 1.5, both tests were able to detect differences at any sample size. For the remainder of the paper, all power scenarios are shown for *δ* = 1. For the Normal distribution, both tests have similar power for all sample sizes. For the Cauchy and Laplace distributions, the WMW has greater power regardless of sample size. The TST has greater power for the Exponential distribution.

**Fig 3:**
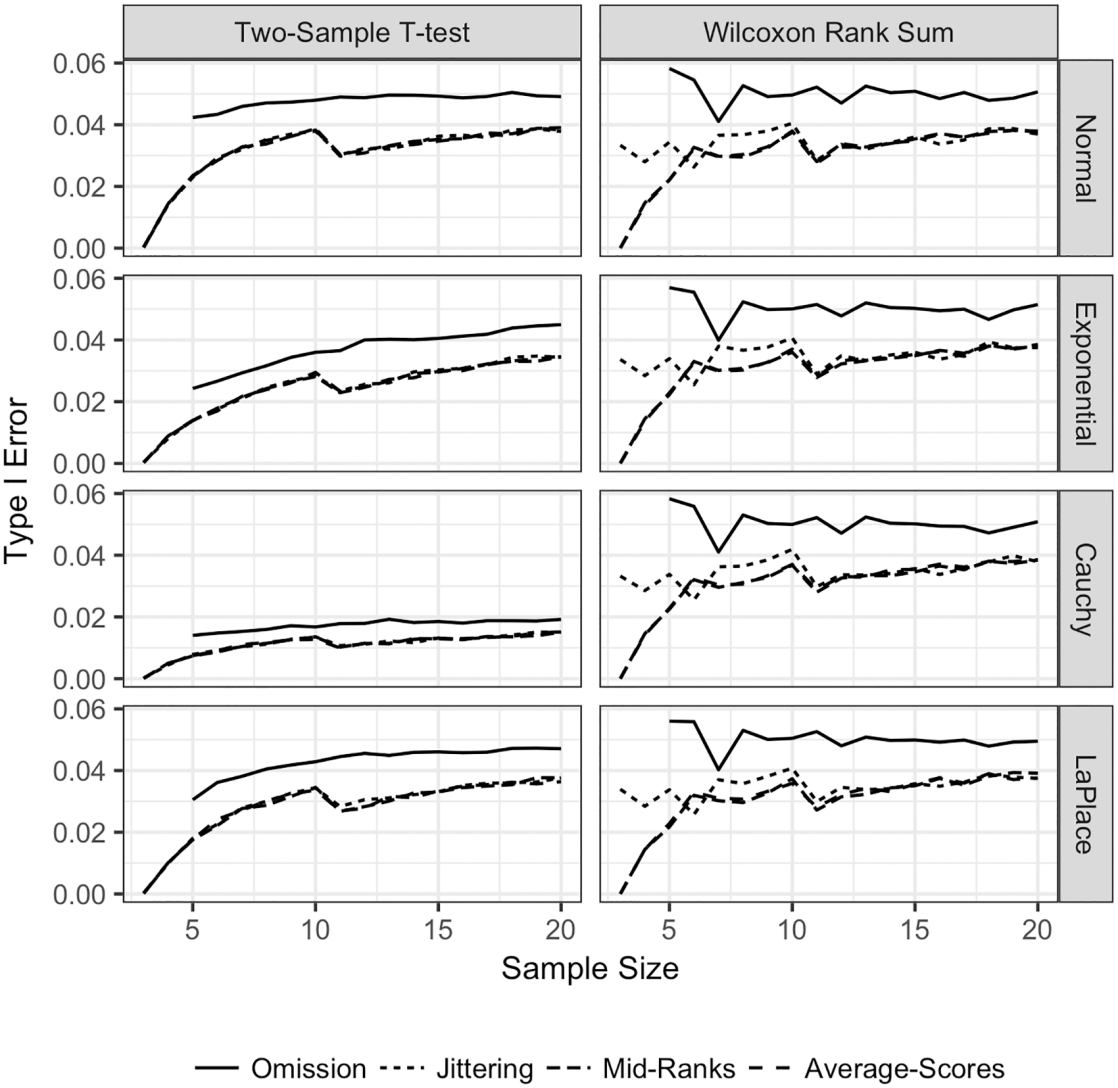
Type I error for the TST and WMW with 10% of observations tied. Type I error for both the TST and WMW with 10% of observations tied, equal sample sizes, and continuous distributions. Type I error is plotted on the vertical axis, and the sample size for one sample is plotted on the horizontal axis. Each line represents the Type I error of a different method of adjusting for ties: omission of tied observations 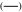, jittering 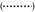, mid–ranks 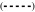, and average–scores 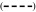. Each row corresponds to a different underlying population distribution: the Normal (top row), exponential (second row), Cauchy (third row) and Laplace (bottom row). Each column corresponds to a different test: the TST on the left and the WMW on the right.

Fig 3 shows the Type I error results for both statistical tests when the sample sizes in the two groups are equal and 10% of the observations are tied. Corrections for ties for WMW are made under one of four scenarios: average–scores, jittering, mid–ranks, and omission. Corrections for ties are not made for TST under the first three scenarios because such corrections are not commonly practiced. Type I error for the t-test on the same data used for WMW is plotted in each instance as a comparison. However, in the fourth scenario, omission of tied ranks, the observations that are tied in TST are omitted, just as they are in WMW. Sample sizes of three and four were not used for the omission scenario, because omission of one observation results in an unrealistically small sample size.

Interestingly, for both tests, the Type I error is closer to nominal for omission of tied observations than it is for any other adjustment. The Type I error remains stable for the WMW, and decreases for the TST. When when *n* = *m* = 3, 4, 5, 6, the jittering method has better Type I error properties than either the mid–rank or average–scores adjustments. This is because the latter two methods result in a decrease in variance, which does not happen when jittering is applied. Once the sample size for each group is greater than seven, the Type I error results are similar, regardless whether one corrects with average-scores, mid-ranks, or jittering. However, the Type I error for omission of observations is always close to *α* = 0.05. Furthermore, the TST seems to require at least 10 observations per sample to have similar Type I error to the WMW, and this observation applies only in the cases of the Normal and Laplace distributions.

For both the TST and WMW, omission of observations when *n* = *m* ≤ 10 results in Type I error closest to the nominal level than any of the other methods for all sample sizes. This is somewhat surprising for the case of the TST, and, as far as is known, has not been previously tested in the literature. As is expected, the Type I error for the WMW is not affected by the underlying distribution. Unexpectedly, Type I error for the TST improves slightly for all underlying distributions when tied observations are omitted. In terms of Type I error, these results show that omission of tied observations gives the best performance for both the TST and the WMW in terms of Type I error for small sample sizes.

**Fig 4:**
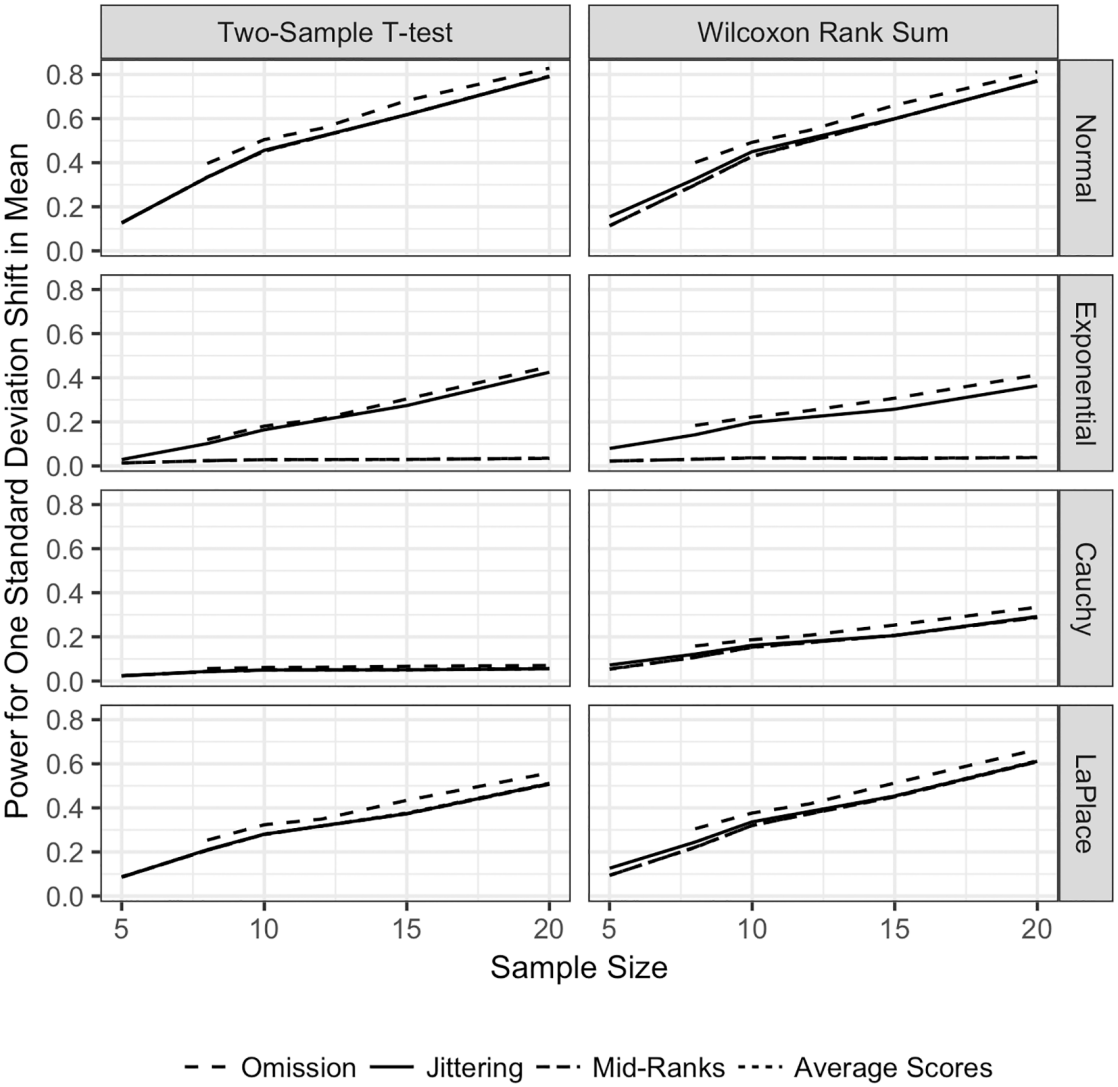
Power for the TST and WMW with 10% of observations tied. Power for both the TST and WMW with 10% of observations tied, equal sample sizes, and continuous distributions. Power is plotted on the vertical axis, and the sample size for one sample is plotted on the horizontal axis. Each line represents the power of a different method of adjusting for ties: omission of tied observations 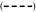, jittering 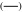, mid–ranks 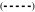, and average–scores 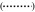. Each row corresponds to a different underlying population distribution: the Normal (top row), exponential (second row), Cauchy (third row) and Laplace (bottom row). Each column corresponds to a different test: the TST on the left and the WMW on the right.

Fig 4 shows the power for *δ* = 1 when 10% of the observations are tied. Results for both the TST and WMW, as well as for all four methods of tie management, are displayed. The distribution of the underlying population has more to do with the power than does either the test or the method of dealing with ties. Both tests have poor power properties for the Cauchy and the exponential distributions, with the WMW performing slightly better in both cases. The WMW has slightly larger power for the Laplace distribution than does the TST under all four tie-correction scenarios.

Surprisingly, the power for omitting tied observations is slightly larger for both the TST and the WMW for all distributions. For example, when *n* = *m* = 10 and the underlying distribution for the data is Normal, the power for the WMW for jittering, average–scores, and mid–ranks is close to 0.4. When the tied ranks are omitted, the power is approximately 0.5. For the same sample size, the jittering method produces a power of approximately 0.45. We conclude that omission or jittering of tied ranks results in Type I error as close or closer to nominal than does adjustment with mid-ranks or average-scores with no sacrifice in terms of statistical power.

### Effect of percentage of tied observations

The previous results are given when 10% of the observations are missing. From this point forward, the effect of increasing percentages of tied observations will be considered. In this paper, results are displayed for the jittering and omission methods. Results for the average–scores and mid–ranks methods can be found in S3 and S4 Figs, respectively.

**Fig 5:**
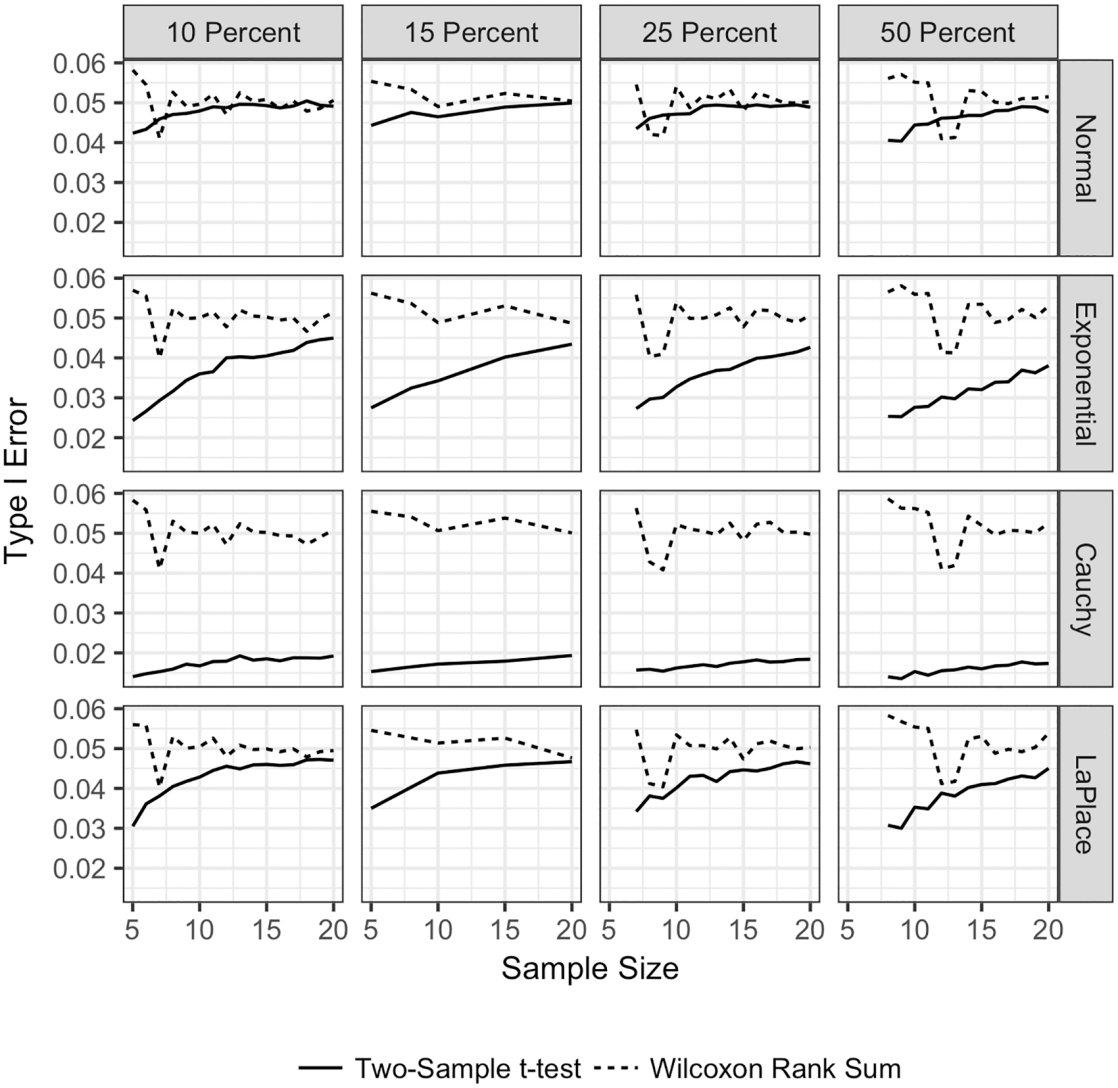
Type I error TST and WMW omitting tied observations. Type I error for both the TST and WMW when tied observations are omitted, when 10% (far left column), 15% (second column), 25% (third column), and 50% (far right column), of the observations are tied; sample sizes are equal; and all distributions are continuous. Type I error is plotted on the vertical axis, and the sample size for one sample is plotted on the horizontal axis. The Type I error for the TST is plotted with a solid line 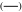, and the Type I error for the WMW is plotted with a dashed line 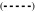 Each row corresponds to a different underlying population distribution: the Normal (top row), exponential (second row), Cauchy (third row) and Laplace (bottom row). Each frame shows the empirical Type I error under each combination of distribution and percentage of tied observations.

Fig 5 shows the Type I error when tied observations are omitted from the data. For the WMW, when there are either 10% or 15% of the observations that are tied., the Type I error is slightly larger than 0.05 when sample sizes are small (*n* = *m* ≤ 7), and tracks closely to the nominal level of *α* = 0.05 for sample sizes larger than seven. When the percentage of tied observations is 25%, the Type I error level has more erratic behavior for the smaller sample sizes, and settles down to the nominal level when *n* = *m* = 10. The Type I error has worse behavior when 50% of the observations are tied. It fluctuates between *α* = 0.04 and *α* = 0.06 until the sample size is 15 in each group. Even such a large number of omitted observations, the WMW has good Type I error properties. As expected, there are very small differences in Type I error level for the WMW among the different distributions.

For the TST, the underlying distribution has more of an impact on the Type I error than does the percentage of tied observations. When the underlying distribution of the data is Normal, which is the best case scenario for the TST, it behaves much like the WMW in terms of Type I error.

**Fig 6:**
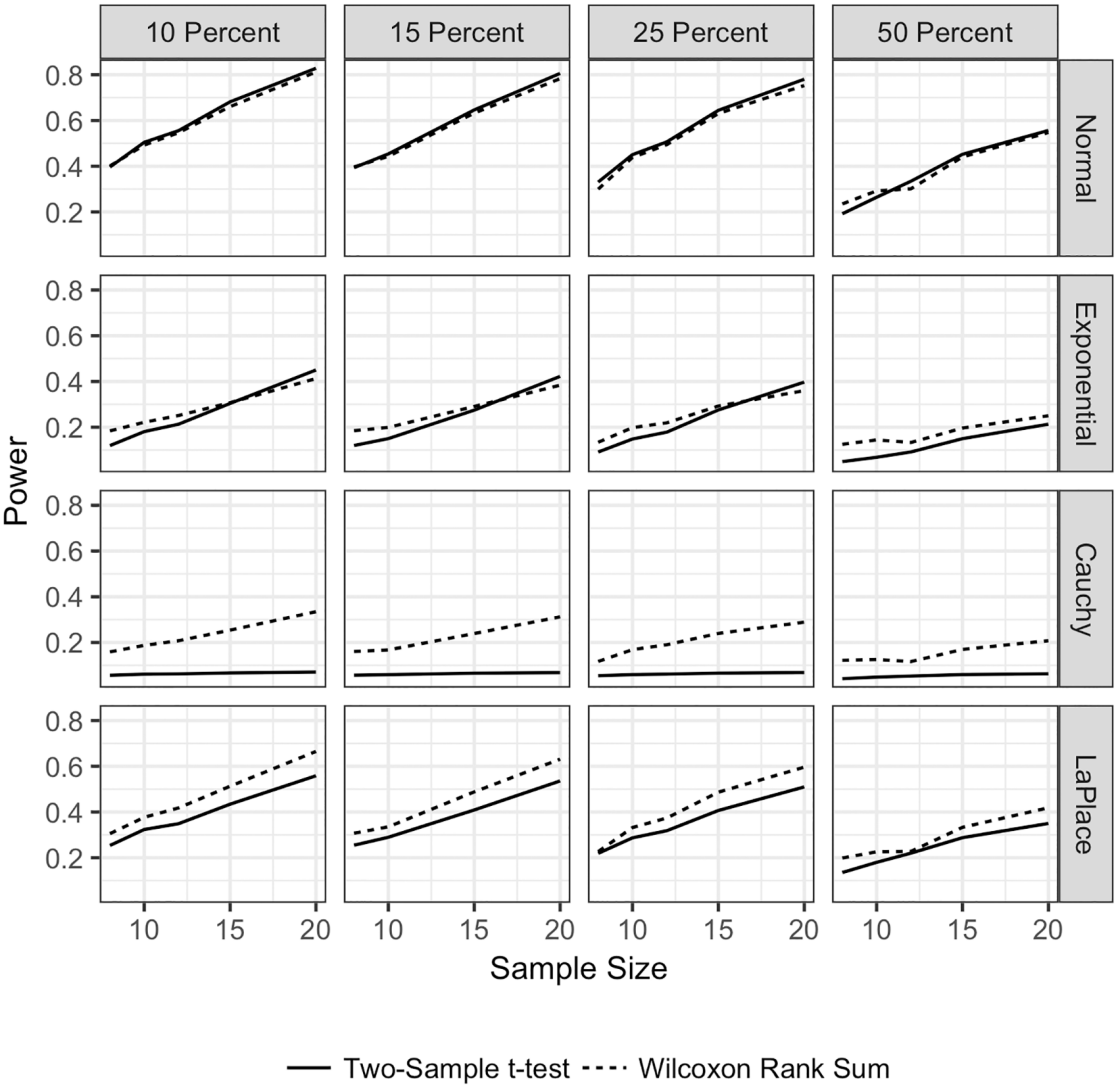
Power for TST and WMW when omitting tied observations. Power for both the TST and WMW when tied observations are omitted, when 10% (far left column), 15% (second column), 25% (third column), and 50% (far right column), of the observations are tied; sample sizes are equal; and all distributions are continuous. Power is plotted on the vertical axis, and the sample size for one sample is plotted on the horizontal axis. The power for the TST is plotted with a solid line 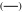, and the power for the WMW is plotted with a dashed line 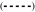. Each row corresponds to a different underlying population distribution: the Normal (top row), exponential (second row), Cauchy (third row) and Laplace (bottom row). Each frame shows the empirical power under each combination of distribution and percentage of tied observations.

Results for power calculations when omitting tied ranks with 10%, 15%, 25%, and 50% tied observations are given in Fig 6. The power is calculated when *δ* = 1. For both the TST and WMW, power levels of 0.8 are obtained only when *n* = *m* = 20 for the 10% and 15% scenarios, and the underlying distribution is Normal. The power is between 0.7 and 0.8 when *n* = 20 for the scenario where 25% of observations are tied. The power is unacceptable for any sample size when 50% of the observations are tied. For distributions other than the Normal distribution, power levels are unacceptable regardless of the percentage of tied observations or the sample size.

**Fig 7:**
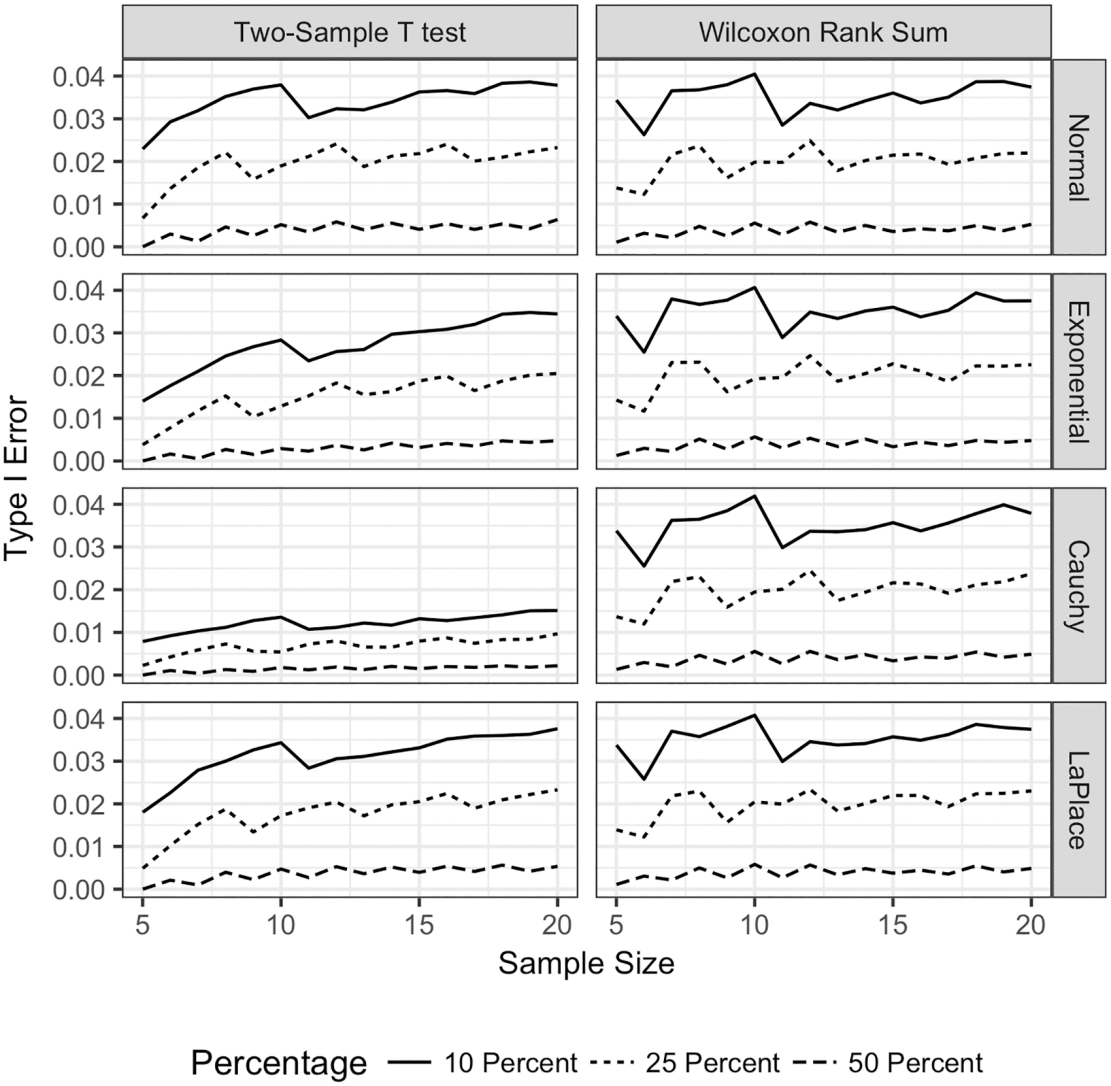
Type I error for TST and WMW with jittering method. Type I error for both the TST and WMW for the jittering method when 10% 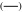, 25% 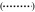, and 50% 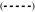, of the observations are tied; sample sizes are equal; and all distributions are continuous. Type I error is plotted on the vertical axis, and the sample size for one of the groups is plotted on the horizontal axis. Each row corresponds to a different underlying population distribution: the Normal (top row), exponential (second row), Cauchy (third row) and Laplace (bottom row). Results for the TST are displayed in the left column; results for the WMW are displayed on the right. Each frame shows the empirical Type I error under each combination of distribution and statistical test.

Fig 7 shows results only for the jittering method of breaking ties among observations when there are 10% (solid line in each frame), 25% (dotted line), and 50% (dashed line) tied observations. Again, tied observations in TST are not corrected. The jittering method produces reasonable results for all sample sizes when 10% tied observations exist. For larger percentages of ties, the Type I error becomes too small. Although having a small Type I error seems appealing, the Type I error is inversely related to Type II error. As the Type I error decreases, the Type II error increases for the same sample size and same variability in the sample. Therefore, the power of the test will decrease.

Table 1 shows the maximum power, which typically corresponds to the largest sample size, for each distribution and each percentage of tied observations when jittering is applied. The power is calculated when *δ* = 1. Power values are unacceptable regardless of distribution or test when there are more than 15% tied observations. Even when there are only 10% ties in the data and the underlying distribution is Normal, the maximum power is still below 0.8, which is typically considered a minimum acceptable power. Therefore, we can conclude that using jittering to adjust for ties in data is only viable for instances when 10% or fewer of the observations are tied. This threshold has been mentioned earlier in this manuscript in reference to other method of adjusting for ties in ranks. Now it has been quantified using jittering adjustment, which has good Type I and Type II error properties in small sample sizes.

**Table 1:** Maximum power for jittering method according to percentage of tied observations.

	TST				WMW			
Distribution	10% Ties	15% Ties	25% Ties	50% Ties	10% Ties	15% Ties	25% Ties	50% Ties
Normal	0.79	0.74	0.34	0.22	0.77	0.72	0.36	0.19
Exponential	0.42	0.38	0.36	0.09	0.72	0.32	0.13	0.06
Laplace	0.51	0.46	0.24	0.11	0.61	0.56	0.76	0.13
Cauchy	0.06	0.05	0.05	0.01	0.29	0.25	0.13	0.04

Maximum power for the medium scenario when jittering is applied to various percentages of tied ranks in the data. Jittering adjustments are not applied to TST.

To this point, results show that (in no particular order):

1. The Type I error and the power of the TST are adversely affected by the presence of ties.
2. Omitting tied observations has the best Type I error and power properties of the four methods of tie adjustment considered, and this effect is true for the TST as well as the WMW.
3. The jittering correction for ties in the WMW has better Type I error properties for very small sample sizes than do either the average-scores or the mid-ranks corrections.
4. Unequal sample sizes have a detrimental effect on Type I error and power for both the WMW and TST, with the effect worsening as the ratio of the sample sizes is larger.
5. Tie correction methods do not have good Type I error and power properties when the percentage of ties in the data is greater than 15%. This effect is somewhat mitigated for sample sizes larger than 20 in each group (*i.e.* a total sample size of 40).
6. The Type I error and power for WMW is not as affected by the underlying distribution are they are for TST.
7. In some situations, the underlying distribution has the greatest effect of any factor that we vary.

Of these seven items, items six and seven are expected results because TST is not robust to the distribution that generated the sample data—one reason that the WMW was developed and has become popular. Therefore, item four is expected to prove true, given that previous experiments show a decrease in power for the TST when sample sizes are unequal. Likewise, item five makes sense, because the variance of the data, which forms the denominator of the TST, decreases in the presence of ties, resulting in a larger test statistic that is more likely to lead to a decision to reject the null hypothesis at the expense of power.

Items one and two are perhaps the most surprising of the results. Omitting tied observations from further analysis seems to result in better Type I error and power properties than any other method considered in this paper. Furthermore, omitting ties has a favorable effect on the TST, as well as the WMW. Item three is also a new result. Replacing ties by jittering does not seem to harm the Type I error or the power of WMW, and it could be helpful when sample sizes are very small. However, the success of jittering does not mean that it should be used in every circumstance because the distributions considered in this paper are continuous. Earlier results have shown that no tie-correction method is recommended when the data have very few possible values, such as in the case of Likert-Scale data [21].

## Conclusion

Omitting tied observations in the WMW test seems to be a valid method of tie adjustment in terms of power and Type I error. The effect is shown not only for the WMW, but also for the TST. In addition, jittering has good Type I error properties for sample sizes as small as *n* = *m* = 3, and the power of the test is at least as good as when more conventional methods are used. However, jittering makes sense only in the context of data that have an underlying continuous distribution.

This observation begs the question of what two-sample test is useful for discrete or ordinal data when ties are present. One method is to use a permutation test, which is a computationally intensive method of calculating the null distribution of the possible difference in means (or medians, or ratios, etc). The use of permutation tests for various designs is described in [28]. Log-linear models and logit models for ordinal data are described in [29]. Although [30] is written for applications in neuroscience, it suggests, particularly in Section 4, methods for analysis of data that are ill-behaved in terms of skewness, heteroscadasticity, outliers, and modality. Methods include comparison of quantiles instead of means and use of modern methods for detecting outliers.

In other words, rank-based methods, although they have their uses, should not be applied in all situations where the data are ill-behaved; rank-based methods were developed under the assumption that the distribution generating the data is continuous, and, in the case of the WMW, that the two populations have the same shape. In such cases, omitting the tied observations seems to be best solution in terms of Type I error and power. The next best option would be jittering the data to take care of tied ranks. In situations where the underlying distribution for the data is discrete, or the data are ordinal, the methods given in [28, 29], and [30] should be considered.

## Supporting information

S1 FigType I error for the TST and WMW with no ties.Each line in the figure represents a different distribution. The Type I error is plotted on the vertical axis, and the sample size for one sample is on the horizontal axis. The four distributions plotted are the Cauchy 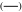, Normal 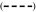, Exponential 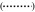, and Laplace 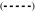. When sample sizes are small, the TST consistently has rejection rates below the nominal level of *α* = 0.05, even for the Normal distribution. Thus, the Type I error for the TST is sensitive to the underlying distribution, and the effect is especially pronounced for the Cauchy distribution (solid line). On the contrary, WMW has a Type I errors close to 0.1 for *n* = 3, which then hovers around the nominal value of 0.05 for all distributions in all sample sizes, except for the Cauchy distribution when *n* = 20, when it behaves more like the TST. Note that the distribution does not have an effect on the WMW with respect to Type I error.(TIFF)

S2 FigPower for the TST and WMW with no ties.Empirical power (1 − *β*) for the TST and WMW and the four distributions in three different scenarios. There are no ties in the data. Each line in the figure represents a different distribution. Power is plotted on the vertical axis, and the sample size for one sample is on the horizontal axis. The four distributions plotted are the Cauchy 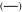, Normal 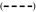, Exponential 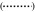, and Laplace 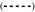. When power is calculated for the TST (WMW) the alternative mean (or the location shift, for the WMW) needs to be specified. The “large” scenario (top row) shows an alternative mean difference, *μ*_*X*_ − *μ*_*Y*_, that is 1.5 standard deviations greater than that of the null mean. In the “medium” scenario (middle row), the alternative mean is one standard deviation from the null mean, and in the “small” scenario (bottom row), the alternative mean is one-half standard deviation from the null mean. The three panels on the left give power under each scenario for TST. The three panels on the right give power for WMW under each alternative shift.(TIFF)

S3 FigType I error for the TST and WMW with 25% tied observations.Type I error versus the sample size for the TST and WMW tests under the scenario that 25% of the observations are tied in the data. Each line in the figure represents a different distribution. Type I error is plotted on the vertical axis. The four distributions plotted are the Cauchy 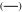, Normal 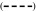, Exponential 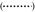, and Laplace 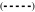. Both tests consistently have rejection rates below the nominal level of *α* = 0.05, even for the Normal distribution. In fact, the maximum value on the vertical scale is 0.025. The Type I error for TST is sensitive to the underlying distribution, and the effect is especially pronounced for the Cauchy distribution (solid line). On the contrary, the distribution does affect WMW with respect to Type I error. The first row of the plot (top two panels) show the Type I error when the ties are adjusted using average–scores. The middle row shows Type I error for a jittering adjustment, and the bottom row shows Type I error for a mid–ranks adjustment.(TIFF)

S4 FigPower for the TST and WMW with 25% tied observations.Empirical power (1 − *β*) for TST and WMW and the four distributions when ties are replaced by average scores or mid-ranks. The power is plotted on the vertical axis, and the sample size for one sample is on the horizontal axis. The top two panels show the power when the average–scores method is used to adjust for ties, and the bottom two panels show the results for the mid–ranks method. The four distributions plotted are the Cauchy 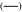, Normal 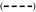, Exponential 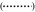, and Laplace 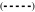. When power is calculated for TST (WMW) the alternative mean (or the location shift, for WMW) needs to be specified. For this graph, the power is calculated under the “medium” scenario, when the alternative mean is one standard deviation from the null mean. The two panels on the left give power under each scenario for TST. The two panels on the right give power for WMW under each power scenario. The maximum power is approximately 60%, and the Normal distribution seems to give the best results for both tests. Neither test performs well for the exponential or the Cauchy, but WMW performs slightly better than the TST for Cauchy distribution. This is expected because the WMW will minimize the effect of outliers that are typical in Cauchy distributions.(TIFF)
