# Supplementary figures and images for: Case for omitting tied observations in the two-sample t-test and the Wilcoxon-Mann-Whitney Test
Source: PLoS One. 2018 Jul 24;13(7):e0200837. doi: 10.1371/journal.pone.0200837 (PMC6057651; doi:10.1371/journal.pone.0200837)

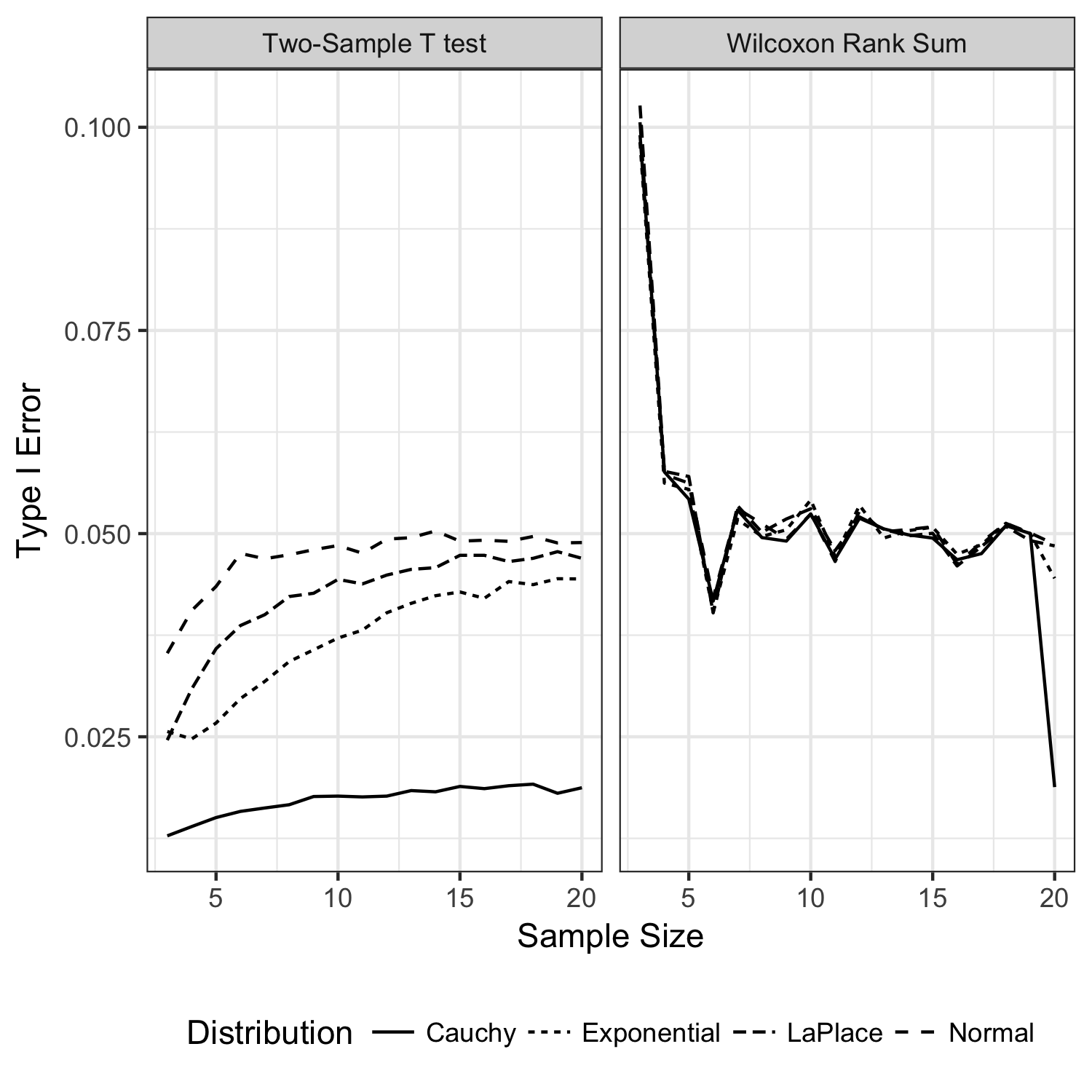

Supplement: S1 Fig — Each line in the figure represents a different distribution. The Type I error is plotted on the vertical axis, and the sample size for one sample is on the horizontal axis. The four distributions plotted are the Cauchy , Normal , Exponential , and Laplace . When sample sizes are small, the TST consistently has rejection rates below the nominal level of α = 0.05, even for the Normal distribution. Thus, the Type I error for the TST is sensitive to the underlying distribution, and the effect is especially pronounced for the Cauchy distribution (solid line). On the contrary, WMW has a Type I errors close to 0.1 for n = 3, which then hovers around the nominal value of 0.05 for all distributions in all sample sizes, except for the Cauchy distribution when n = 20, when it behaves more like the TST. Note that the distribution does not have an effect on the WMW with respect to Type I error. (TIFF) [file pone.0200837.s001.tiff]

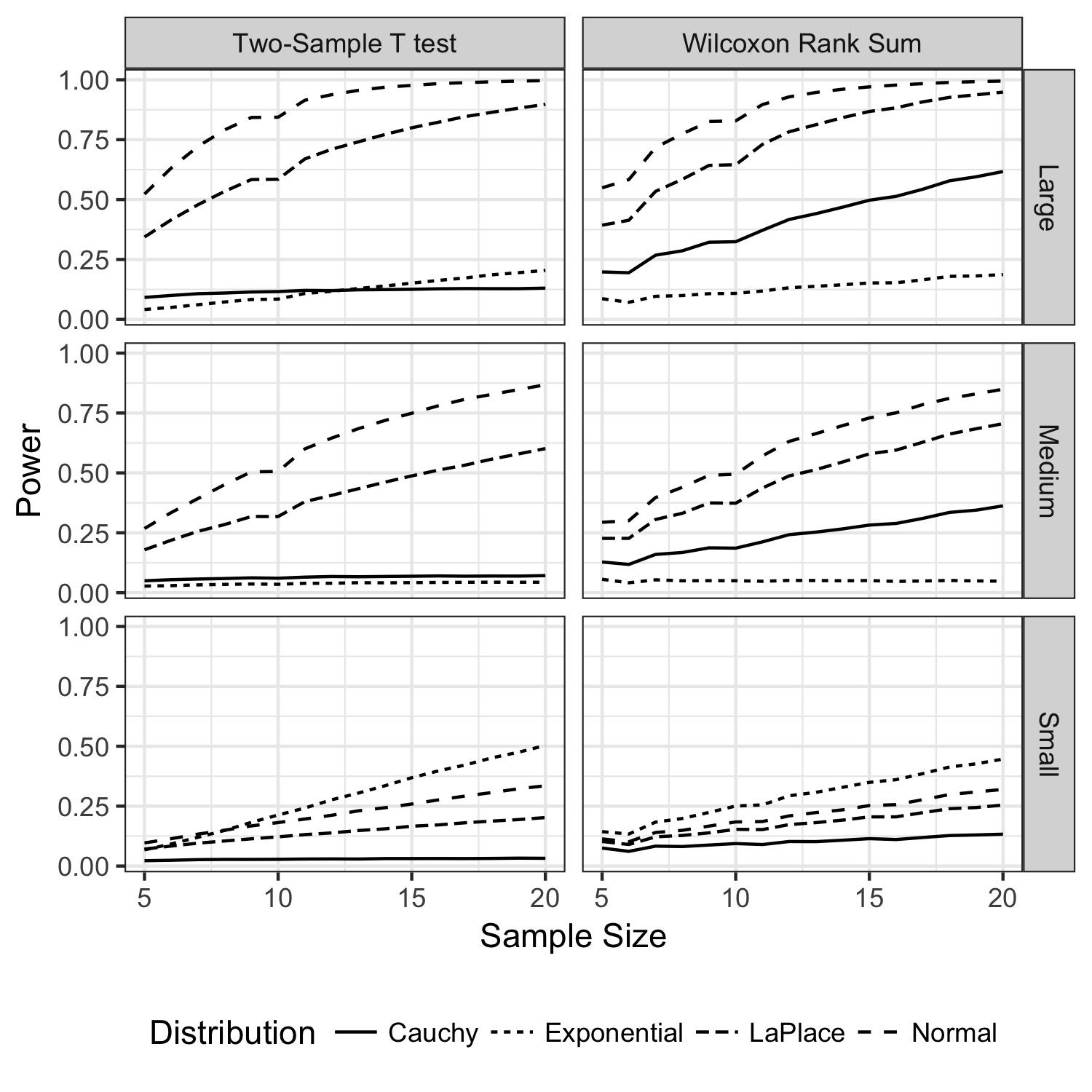

Supplement: S2 Fig — Empirical power (1 − β) for the TST and WMW and the four distributions in three different scenarios. There are no ties in the data. Each line in the figure represents a different distribution. Power is plotted on the vertical axis, and the sample size for one sample is on the horizontal axis. The four distributions plotted are the Cauchy , Normal , Exponential , and Laplace . When power is calculated for the TST (WMW) the alternative mean (or the location shift, for the WMW) needs to be specified. The “large” scenario (top row) shows an alternative mean difference, μX − μY, that is 1.5 standard deviations greater than that of the null mean. In the “medium” scenario (middle row), the alternative mean is one standard deviation from the null mean, and in the “small” scenario (bottom row), the alternative mean is one-half standard deviation from the null mean. The three panels on the left give power under each scenario for TST. The three panels on the right give power for WMW under each alternative shift. (TIFF) [file pone.0200837.s002.tiff]

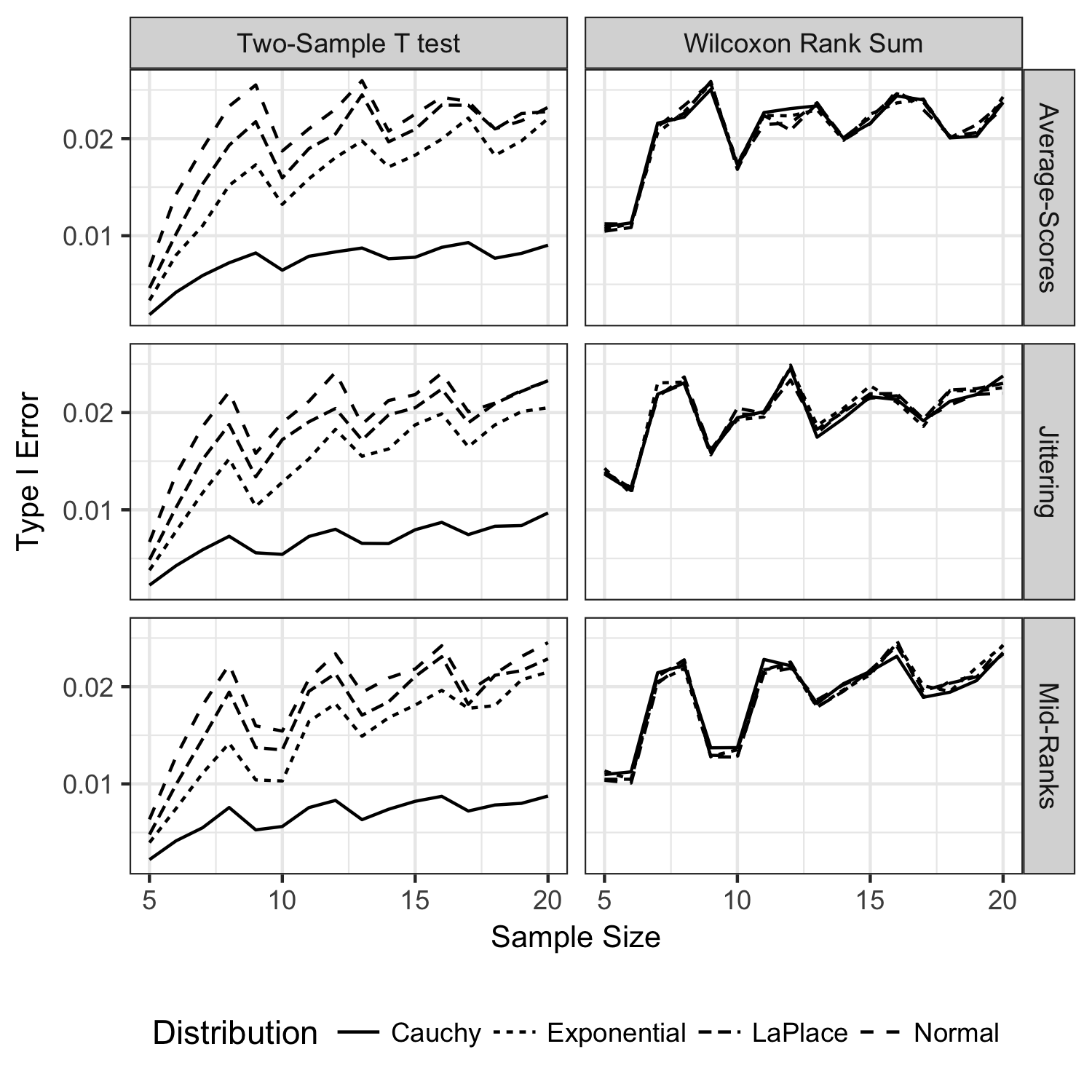

Supplement: S3 Fig — Type I error versus the sample size for the TST and WMW tests under the scenario that 25% of the observations are tied in the data. Each line in the figure represents a different distribution. Type I error is plotted on the vertical axis. The four distributions plotted are the Cauchy , Normal , Exponential , and Laplace . Both tests consistently have rejection rates below the nominal level of α = 0.05, even for the Normal distribution. In fact, the maximum value on the vertical scale is 0.025. The Type I error for TST is sensitive to the underlying distribution, and the effect is especially pronounced for the Cauchy distribution (solid line). On the contrary, the distribution does affect WMW with respect to Type I error. The first row of the plot (top two panels) show the Type I error when the ties are adjusted using average–scores. The middle row shows Type I error for a jittering adjustment, and the bottom row shows Type I error for a mid–ranks adjustment. (TIFF) [file pone.0200837.s003.tiff]

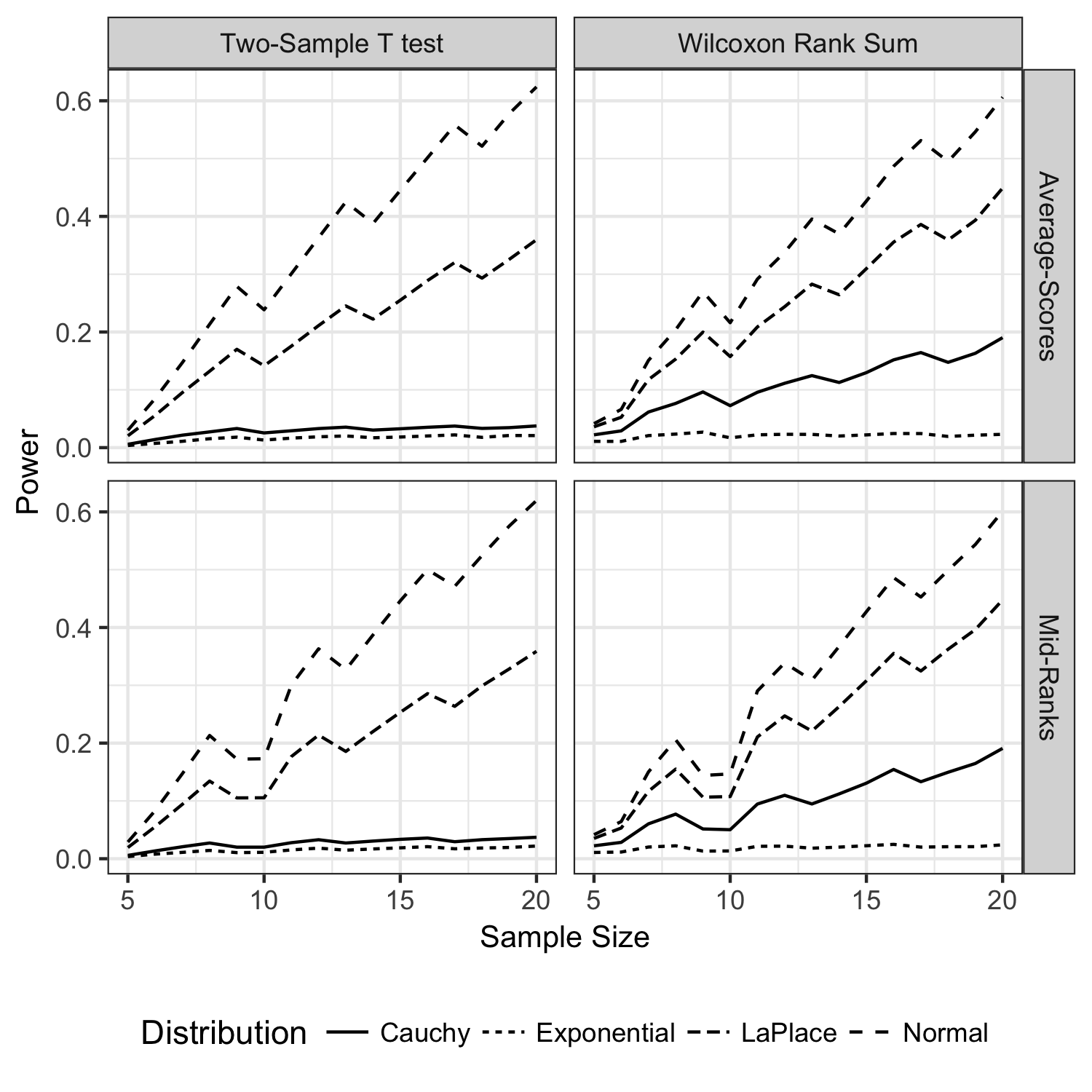

Supplement: S4 Fig — Empirical power (1 − β) for TST and WMW and the four distributions when ties are replaced by average scores or mid-ranks. The power is plotted on the vertical axis, and the sample size for one sample is on the horizontal axis. The top two panels show the power when the average–scores method is used to adjust for ties, and the bottom two panels show the results for the mid–ranks method. The four distributions plotted are the Cauchy , Normal , Exponential , and Laplace . When power is calculated for TST (WMW) the alternative mean (or the location shift, for WMW) needs to be specified. For this graph, the power is calculated under the “medium” scenario, when the alternative mean is one standard deviation from the null mean. The two panels on the left give power under each scenario for TST. The two panels on the right give power for WMW under each power scenario. The maximum power is approximately 60%, and the Normal distribution seems to give the best results for both tests. Neither test performs well for the exponential or the Cauchy, but WMW performs slightly better than the TST for Cauchy distribution. This is expected because the WMW will minimize the effect of outliers that are typical in Cauchy distributions. (TIFF) [file pone.0200837.s004.tiff]
